# Dispositional Mindfulness and Self-Compassion Buffer the Effects of COVID-19 Stress on Depression and Anxiety Symptoms

**DOI:** 10.1007/s12671-022-02008-0

**Published:** 2022-10-22

**Authors:** Shadi Beshai, Saba Salimuddin, Nabhan Refaie, Jenna Maierhoffer

**Affiliations:** 1 Department of Psychology, University of Regina, Regina, Saskatchewan Canada; 2 Gordon S. Lang School of Business and Economics, University of Guelph, Guelph, Ontario Canada

**Keywords:** Self-compassion, Dispositional mindfulness, COVID-19, Moderation, Resilience, Depression, Anxiety

## Abstract

**Objectives:**

The COVID-19 pandemic has been associated with a dramatic rise in symptoms of depression and anxiety. Dispositional mindfulness (DM) and self-compassion (SC) have consistently been associated with psychological disorder symptoms and appear to buffer the effects of stress on depression and anxiety.

**Methods:**

Across two studies (*n* = 888), we examined direct and indirect (moderation) relationships of DM, SC, COVID-19-related stress, and symptoms of depression and anxiety. We also examined the differential effects of several DM measures (FFMQ-15; FFMQ-39; MAAS) in the relationships of COVID-19 stress and psychological disorder symptoms. We recruited participants (Study 1 *n* = 350; 42.2% cis women; Study 2 *n* = 538; 44.3% cis women) online (MTurk) and examined associations of DM, SC, and COVID-19 stress, and emotional impact, and the moderating effect of DM and SC in the relationships of COVID-19-related fears, stress, emotional impacts, and psychological disorder symptoms.

**Results:**

DM and SC were moderately and negatively correlated with COVID-19 fears and stress (correlations ranging *r* =  − .14 to *r* =  − .42) across studies. Study 1 moderation analyses demonstrated SC, but not DM (FFMQ-15), significantly moderated relationships of COVID-19 fears and emotional impacts with symptoms. Study 2 analyses demonstrated the FFMQ-39, but not the MAAS, significantly moderated relationships of COVID-19 stress and psychological disorder symptoms.

**Conclusions:**

These results support the potential protective roles of DM and SC in disrupting pathological trajectories related to naturally elevated pandemic stress. Results also demonstrate the differential associations of several DM measures with COVID-19 stress. Future research should replicate such findings with more diverse samples and using various measures of self-compassion and risk metrics.

**Supplementary Information:**

The online version contains supplementary material available at 10.1007/s12671-022-02008-0.

Prior to the pandemic, there was a trend of deteriorating mental health among members of the general population (Andersen et al., 2011). Unfortunately, the COVID-19 crisis has exacerbated this trend (Vindegaard & Benros, 2020). Evidence suggests heightened symptoms of depression among 14.6 to 48.3% of the general population in several nations around the world during the pandemic (Xiong et al., 2020). This increase in depression symptoms can be explained by several factors, such as increased perceived stress, fear, uncertainty, and the socio-political instability that was instigated by the crisis (Talevi et al., 2020). Depression is defined as the experience of a constellation of symptoms—depressed mood; anhedonia; changes in sleep or eating patterns; fatigue; thoughts of worthlessness; suicidal ideation—for two weeks or longer that cause significant distress or interruption to daily living (APA, 2013). Anxiety is a broad diagnostic category, typified by symptoms of apprehension, worry, and fear, which are often accompanied by somatic symptoms (e.g., muscle tension; sleep disturbances; restlessness; rapid breathing; trembling; fatigue; APA, 2013). Depression is the leading cause of disability worldwide (Friedrich, 2017). Similarly, anxiety symptoms and conditions are associated with enormous costs at the societal and individual levels (Lépine, 2002). Accordingly, elucidating the risk and protective mechanisms of anxiety and depression, especially during the throes of a global crisis such as COVID-19, is incredibly worthwhile from humanitarian and practical perspectives.

Although there is no consensus on the definition of resilience, it is often defined as the ability to recover, “bounce back,” or cope despite the presence of substantial adversity (Earvolino-Ramirez, 2007; Rutter, 1965). First, this definition of resilience suggests mental health, including the severity of depression and anxiety symptoms, is the by-product of the transaction between risk and protective factors. Second, it suggests two routes to mental health maintenance: (a) through the reduction or (highly improbable) complete elimination of adversity, stress, or dismantling of other risk factors known to increase the likelihood of ill health; and/or (b) through the cultivation of protective mechanisms known to offer resilience or help people cope *despite* the presence of stress and other risk factors exacerbating pathology. Ideally, both routes are engaged in the path toward recovery; however, and given the ever-present stress of modern living—now exacerbated by the uncertainty and grief brought on by the COVID-19 crisis—fostering protective factors may be a more realizable path toward positive mental health outcomes.

A large and growing body of evidence is supporting the protective effects of Buddhism-inspired concepts such as mindfulness and self-compassion (Shonin et al., 2014). Accordingly, both concepts and their applications in clinical practice may offer ways to cultivate resilience during the pandemic. Much like the concept of resilience, there is still no widely accepted definition or operationalization of mindfulness (Chiesa, 2013; Van Dam et al., 2018). However, the concept is often defined as purposeful awareness of present-moment experiences with an attitude of openness, acceptance, and balance (Kabat-Zinn, 1994). Dispositional mindfulness (DM) is the capacity to be mindful in daily life, which is believed to be normally distributed and naturally occurring in the general population (Brown & Ryan, 2003). That is, there is a consensus that DM is conceptually different from cultivated or trained forms of mindfulness (Rau & Williams, 2016).

Self-compassion (SC) is defined as being aware of one’s own suffering, and having the desire or motivation to alleviate this suffering for self (Neff, 2003a). Neff was the first to operationalize self-compassion and argued that it is composed of three-dimensional factors. The first dimension is self-kindness, which stands in opposition to self-criticism and judgement, while the second is mindfulness of one’s own suffering, an awareness qualitatively different from over-identification with and/or avoidance of suffering. The third dimension of Neff’s (2003a) conceptualization of SC is the recognition of the common humanity of suffering, as opposed to the perception of isolation from others in times of suffering. Neff’s original conceptualization of self-compassion envisioned this capacity as a way to buffer the effects of stress against the development of heightened symptoms of depression and anxiety (Bluth & Neff, 2018; Neff, 2003a, b).

Evidence suggests both DM and SC are consistently inversely associated with symptoms of depression, anxiety, and other forms of psychopathology (Van Dam et al., 2011). Second, both such protective dispositions are inversely associated with vulnerability factors implicating depression and anxiety, including but not limited to rumination (Odou & Brinker, 2014; Raes & Williams, 2010), perfectionism (Ferrari et al., 2018), cognitive biases (Hanley et al., 2015), mind-wandering (Greenberg et al., 2018), and intolerance of uncertainty (Mantzios et al., 2015).

Third, and pertinently, both DM and SC buffer or moderate relationships between experiences of adversity or stress and psychopathology. For example, DM moderated the relationships of stress and dysphoric mood among adolescents (Ciesla et al., 2012) and moderated the relationship of reported experience of childhood maltreatment and chronicity of depression over time (Beshai & Parmar, 2018). DM was also demonstrated to moderate the relationship between neuroticism and depression symptoms (Barnhofer et al., 2011), and the relationship between the experience of trauma and PTSD symptoms (Tubbs et al., 2019). In all the above-cited examples, higher levels of DM attenuated associations of risk factors and symptoms of psychological disorders. Consistent with this, emerging evidence is suggesting DM significantly moderates the relationship between COVID-19 stress and psychological disorder symptoms (Conversano et al., 2020; Liu et al., 2022; Royuela-Colomer et al., 2022). Conversano et al. (2020) found DM to be the most important predictor of psychological distress among those experiencing stressful experiences in response to the pandemic. Saricali et al. (2020) found DM mediated the relationship between COVID-19 fears and hopelessness. Finally, researchers found DM was associated with COVID-19-related stress, worry, depression, and positive coping (Dillard & Meier, 2021).

Not surprisingly, researchers also found SC to play a protective role during the COVID-19 crisis. SC was positively and significantly associated with life satisfaction among 337 self-quarantining Chinese participants (Li et al., 2021). SC was also negatively correlated with perceived COVID-19 threat among Turkish community adults (Kavakli et al., 2020), and negatively correlated with intolerance of uncertainty and COVID-19 fears among city-dwelling participants in Turkey (Deniz, 2021). SC moderated the relationship between perceived threats and psychological distress during the pandemic among people living in Hong-Kong (Hi-Po Lau et al., 2020). In another recent study with over 4000 participants from 21 countries, compassion for self, others, and from others moderated relationships between perceived threat of COVID-19 and depression, anxiety, and perceived stress (Matos et al., 2022). Accordingly, both and SC appear to directly or indirectly associate with COVID-19-related stressors and fears.

Several mechanisms have been proposed to explicate the buffering effects of DM and SC. High levels of DM are associated with heightened neural activation in brain regions associated with self-referential thinking and self-regulation, while associated with attenuated neural activation in brain regions associated with emotional reactivity even in the context of viewing emotional stimuli (Kong et al., 2016; Zeidan et al., 2018). Accordingly, it is conceptualized that DM works to buffer the effects of risk on symptoms of psychological disorders through the synergy of improved self-regulation and reduced emotional reactivity to stressful or emotional content (Rau & Williams, 2016). SC is likely to exert its buffering effect by tapping ancient care-seeking, care-giving, nurturance, and empathy-related brain circuitry (Hermanto & Zuroff, 2016). For example, evidence suggests compassion more generally is associated with a specific caregiving psychological profile evolutionarily positioned to help ease others’ distress and protect them from suffering (Goetz et al., 2010).

Unfortunately, there are currently very few studies that have examined the buffering effects of DM and SC in the relationships of COVID-19 stress and symptoms of depression and anxiety. Furthermore, no published investigations to date have compared the differential moderation effects of DM as measured by several validated measures of the construct. As outlined, there is mounting evidence suggestive of this buffering effect of DM and SC. Second, skills related to DC and SC are readily cultivated, even using brief and scalable interventions (Beshai et al., 2020) designed specifically for this purpose.

The above-reviewed literature suggests (a) depression and anxiety symptoms have unfortunately risen on a global level during the COVID-19 pandemic; (b) consistent with theories of psychological resilience, protective factors offer a way to mitigate exacerbating effects of stressors and other forms of adversity; (c) DM and SC are attractive protective factors, since extant evidence and theories support their direct and indirect effects in reducing depression and anxiety. In this two-study investigation, we sought to examine how DM, as assessed through three commonly used and validated scales, and SC (a) directly correlate with COVID-19 stress and impacts; and (b) moderate the relationships between COVID-19 stress, impacts, and symptoms of depression and anxiety.

## Study 1

In study 1, we sought to examine the relationships of dispositional mindfulness (DM) and dispositional self-compassion (SC) with COVID-19 fears and impacts. We also examined whether greater levels of DM and/or SC would buffer the association of COVID-19 fears and depression and anxiety symptoms. We hypothesized that DM and SC would (H1a) significantly and negatively correlate with COVID-19 fears, and depression and anxiety symptom severity; and (H1b) moderate the relationship between COVID-19 fears and depression and anxiety symptoms.

## Method

### Participants

Four hundred and eighty-three participants were recruited from TurkPrime, an extension of Amazon’s Mechanical Turk (MTurk; Litman et al., 2017). Participants were compensated with 2.00 USD for their time, which is commensurate with compensation in other crowdsourcing studies (Chander & Shapiro, 2016). A total of 133 participants were excluded for failing the included attention check items (*n* = 38), and for withdrawing consent to include their data in the final analyses (*n* = 95). The final sample included 350 participants (199 cis men; 148 cis women, 2 non-binary identified, 1 gender not disclosed; *M*_*age*_ = 36.68, *SD*_*age*_ = 11.10). Other sample demographics are presented in Table 1. Prior to the commencement of any study activities, this study was approved by the University of Regina’s Research Ethics Board (#2019–214). Data collection took place in May 2020.

**Table 1:** Sample demographics

Demographics	Study 1 (%)	Study 2 (%)
Ethnicity*		
White	80.9	78.8
Asian	8.6	7.1
Indigenous	-	0.4
Black	12.3	7.1
Middle Eastern	-	0.2
Hispanic	4.6	5.4
Other/unknown	3.1	1.1
Education		
High school (or equivalent)	19.7	22.1
Trades or other certificate or diploma	-	20.1
College/University or diploma above bachelor’s level	62.3	46.2
Degree in medicine, dentistry, veterinary medicine, optometry	-	0.4
Post-graduate	17.7	12.3
Unknown	0.3	-
Marital status		
Single, never married	29.1	47.4
Currently dating, not cohabiting	6.9	-
Married or cohabiting	58.3	41.4
Divorced or separated	4.9	9.9
Widowed	0.9	1.3
Employment status		
Full-time employed	81.1	-
Part-time employed	8.9	-
Unemployed	7.1	-
Retired	2.9	-
Personal annual income		
Unemployed/No yearly income	-	4.1
Less than $10,000	8.6	0.6
$10,000-$30,000	26.9	27.0
$30,001-$50,000	30.7	27.5
$50,001-$75,000	19.5	21.7
$75,001-$100,000	10.6	12.5
Greater than $100,000	3.7	6.7
Unknown	0.3	-
Household annual income		
Less than $10,000	2.9	-
$10,000-$30,000	14.6	-
$30,001-$50,000	26.6	-
$50,001-$75,000	26.6	-
$75,001-$100,000	15.1	-
Greater than $100,000	14.3	-

^*^Participants could select multiple options to describe their ethnic background in Study 1

### Procedures

Participants first provided consent to participate in the study. Then, they completed demographic items, followed by the SCS and FFMQ in a random order. They then completed the FCV-19S, C19-IS, PHQ-9, and GAD-7 in a random order. Lastly, participants were debriefed and compensated for their participation.

### Measures

#### Patient Health Questionnaire – 9

*Patient Health Questionnaire – 9 (PHQ-9;* Kroenke et al., 2001) is a 9-item self-report scale measuring depressive symptoms. Participants are asked how often they have experienced depressive symptoms (e.g., “low mood or depression; “Poor appetite or overeating”) over a 2-week period using a 4-point Likert scale, ranging from 0 (*not at all*) to 3 (*nearly every day*). Higher total scores on the PHQ-9 are indicative of greater distress. The PHQ-9 was able to distinguish between those diagnosed with major depression and those not diagnosed (Udedi et al., 2019), and higher PHQ-9 scores were associated with higher scores on other depression self-report scales and lower perceived health scores (Martin et al., 2006).

#### Generalized Anxiety Disorder – 7

*Generalized Anxiety Disorder – 7 (GAD-7;* Spitzer et al., 2006) is a 7-item self-report scale measuring general anxiety disorder symptoms. Participants are asked how often they have experienced anxiety symptoms (e.g., “Trouble relaxing”) over a 2-week period using a 4-point Likert scale, ranging from 0 (*not at all*) to 3 (*nearly every day*). GAD-7 scores were able to distinguish between those diagnosed with anxiety disorders and those not diagnosed (Zhong et al., 2015), and were significantly associated with higher scores on other anxiety self-report scales and greater visits to primary care (Ruiz et al., 2011).

#### Fear of COVID-19 Scale

*Fear of COVID-19 Scale (FCV-19S;* Ahorsu, et al., 2020) is a 7-item self-report scale measuring fearful responses to the COVID-19 pandemic. Participants are asked to indicate the extent to which they agree with several self-describing statements indicating a fearful response to COVID-19 (e.g., “My hands become clammy when I think about COVID-19”), using a 5-point Likert scale ranging from 1 (*Strongly Disagree*) to 5 (*Strongly Agree*). Researchers have found the FCV-19S to be a reliable instrument of COVID-19 fear (Bitan et al., 2020). Scores on the FCV-19S have been positively associated with psychological distress and negatively associated with life satisfaction (Satici et al., 2021).

#### COVID-19 Impact Scale

We developed a 5-item bespoke self-report scale (COVID-19 Impact Scale or C19-IS) to measure negative feelings resulting from the perceived social and economic impacts of the COVID-19 pandemic. Participants were asked to indicate the extent to which they experience negative emotional reactions to COVID-19-related impacts (e.g., “I am sad about all the problems and loss the coronavirus has caused”), using an 11-point sliding scale, ranging from 0 (*Not at all how I currently feel*) to 10 (*Exactly how I currently feel*). Although no study other than the current one has used the C19-IS, the scale demonstrated good internal consistency (Table 2) and correlated significantly with the FCV-19S (see Table 3).

**Table 2:** Measures descriptive statistics, reliabilities, and missing data rates

	Mean (SD)	Cronbach alpha (*α*)	McDonald’s omega (ω)	Missing data rate (%)
PHQ-9	7.84 (6.80)	.92	.92	0.00
GAD-7	13.60 (5.50)	.91	.91	0.00
FCV-19S	2.76 (1.03)	.91	.90	0.00
C19-IS	5.63 (2.33)	.83	.84	0.00
FFMQ-15	50.38 (6.39)	.76	.65	0.00
SCS	3.19 (0.62)	.94	.93	0.00

*PHQ-9*, Patient Health Questionnaire – 9; *GAD-7*, Generalized Anxiety Disorder – 7; *FCV-19S*, Fear of COVID-19; *C19-IS*, COVID-19 Impact Scale; *FFMQ-15*, Five Factor Mindfulness Questionnaire – 15; *SCS*, Self-Compassion Scale; *SD*, standard deviation; *α*, Cronbach’s alpha

#### Five-Factor Mindfulness Questionnaire – 15

*Five-Factor Mindfulness Questionnaire – 15 (FFMQ-15;* Gu et al., 2016) is a 15-item self-report scale measuring five facets of mindfulness: Description, Observation, Acting with Awareness, Nonjudgment, and Nonreaction. Participants are presented with a set of self-describing statements (e.g., “I’m good at finding words to describe my feelings”), and are asked to indicate the extent to which these statements represent them or not using a 5-point Likert scale ranging from 1 (*never or very rarely true*) to 5 (*very often or always true*). Those scoring high on the FFMQ-15 have a lower likelihood of a history of major depression (Asensio-Martinez et al., 2019) and have higher well-being scores (Goldberg et al., 2016).

#### Self-Compassion Scale

*Self-Compassion Scale (SCS;* Neff, 2003a, b) is a 26-item self-report scale measuring dispositional self-compassion. Participants are asked to indicate how often they engage in a set of actions reflecting self-compassion (e.g., “I’m kind to myself when I’m experiencing suffering”), using a 5-point Likert scale ranging from 1 (*Almost Never*) to 5 (*Almost Always*). The SCS contains six subscales with four items each: self-kindness, self-judgment (reverse-scored), isolation (reverse-scored), mindfulness, over-identification (reverse-scored), and common humanity. In this study, we computed a dispositional self-compassion score by averaging all subscale items. Past research has shown that self-compassion training increases SCS scores, and SCS scores are negatively related to depression and anxiety symptoms (Cunha et al., 2016).

Measure descriptives, reliabilities, and missing data rates are presented in Table 2.

### Data Analyses

To detect medium moderation effects, sample sizes of over 300 participants are often recommended (Aguinis et al., 2017). We conducted a preliminary data check to evaluate violations of assumptions of normality and the influence of common method variance (e.g., Harman’s single-factor test, Harman, 1960). To test H1a, we conducted zero-order correlations between scores on the FFMQ-15, SCS; FCV-19S, C19-IS, and scores on the PHQ-9 (depression) and GAD-7 (anxiety). We tested H1b by employing Hayes’ (2017) PROCESS SPSS macro (model 1). Models were only tested if there were significant correlations between all components of that model (e.g., FCV-19S, FFMQ, and PHQ-9 scores were all significantly intercorrelated, the moderation model including these variables was tested). Wherein a moderation effect was significant, we provided conditional effects of this moderation. All analyses were conducted using SPSS (V23), and the alpha was set to 0.05.

## Results

### Preliminary Data Checks

Skewness ranged from − 0.54 (C19-IS) to 0.52 (FFMQ-15), and kurtosis ranged from -1.34 (PHQ-9) to 1.1 (SCS). These values were all within acceptable ranges (Cain et al., 2017). Harman’s single-factor test was not significant, accounting for only 31.9% of the variance.

### Zero-Order Correlations

Table 3 summarizes correlation coefficients of the relationships of COVID-19 fears and perceived emotional impacts, DM and SC, and psychological disorder symptoms. Scores on the FFMQ-15 (DM) and SCS (SC) were significantly negatively associated with scores on the FCV-19S (COVID-19 fears), C19-IS (COVID-19 emotional impact), PHQ-9 (depression), and GAD-7 (anxiety). We conducted Fisher’s r-to-z transformations to examine whether the relationships between DM and COVID-19 outcomes were significantly different from the relationships between SC and COVID-19 outcomes. DM had a greater negative relationship with COVID fears (FCV-19S) compared to the relationship between SC and COVID fears (*Z* = 1.68, *p* = 0.047). However, there was no significant difference in the degree of association of DM scores and COVID-19 emotional impact (C19-IS), SC scores with the same measure (*Z* = 0.98, *p* = 0.164).

**Table 3:** Correlations between COVID-19 cognitions, emotional regulation strategies, and mental health symptoms

	FCV-19S	C19-IS	FFMQ-15	SCS-SF	PHQ-9
C19-IS	.45***	-			
FFMQ-15	− .42***	− .20***	-		
SCS	− .31***	− .27***	.61***	-	
PHQ-9	.61***	.35***	− .53***	− .52***	-
GAD-7	.63***	.37***	− .49***	− .54***	.84***

*** = *p* < .001. *FCV-19S*, Fear of COVID-19 Scale; *C19-IS*, COVID-19 Impact Scale; *FFMQ-15*, Five Factor Mindfulness Questionnaire – 15; *SCS*, Self-Compassion Scale; *PHQ-9*, Patient Health Questionnaire – 9; *GAD-7*, Generalized Anxiety Disorder – 7

### Moderating Effect of Mindfulness on Relationship between COVID-19 Fears, Impacts, and Psychological Disorder Symptoms

We tested the moderation effects of FFMQ-15 scores on the relationships between scores on FCV-19S, C19-IS, PHQ-9, and GAD-7, respectively (Supplementary Materials—Table S1). In both cases, the moderating effect of DM was not significant. Similarly, FFMQ-15 scores did not significantly moderate the relationships of C19-IS scores on depression nor anxiety symptoms (Supplementary Materials—Table S1).

### Moderating Effect of Self-Compassion on the Relationship between COVID-19 Fears, Impacts, and Psychological Disorder Symptoms

As shown in Table 4, SCS scores (SC) significantly moderated the relationship between COVID-19 fears and depressive symptoms. As shown in Fig. 1 (Supplemental Materials), compared to those low in SC, highly self-compassionate individuals had significantly lower depression scores compared to those low and moderate in self-compassion when COVID-19 fears were highest (see Table 4 for conditional effects of COVID-19 fears on depression symptoms at different levels of SC). These differences were attenuated when COVID-19 fears were low. Furthermore, moderately self-compassionate individuals had slightly lower scores on the depressive symptoms measure than those low in SC when COVID-19 fears were moderate or high compared to low levels of COVID-19 fears. A similar trend occurred for anxiety symptoms (see Table 4 and Supplemental Materials—Fig. 2).

**Table 4:** Moderating effect of self-compassion on relationships of COVID-19 fears

COVID-19 fears and depression					
	*B*	*SE*	*t*	*p*	*Δr*^*2*^
Model summary	*F*(3, 346) = 116.84, *p* < .001, *r*^*2*^ = .50				
Constant	7.63	0.27			
FCV-19S	3.29	0.26	12.51	< .001	
Self-compassion	− 4.57	0.50	− 9.22	< .001	
FCV-19S x self-compassion	− 1.12	0.46	− 2.43	.016	.009
Conditional effects					
Self-compassion	*B*	*SE*	*t*	*p*	95% CI
Low	3.76	0.32	11.68	< .001	[3.13, 4.39]
Medium	3.39	0.27	12.80	< .001	[2.87, 3.91]
High	2.51	0.42	5.95	< .001	[1.68, 3.34]
COVID-19 fears and anxiety					
	*B*	*SE*	*t*	*p*	*Δr*^*2*^
Model summary	*F*(3, 346) = 143.32, *p* < .001, *r*^*2*^ = .55				
Constant	13.32	0.21			
FCV-19S	2.72	0.20	13.51	< .001	
Self-Compassion	− 4.15	0.38	− 10.92	< .001	
FCV-19S x Self-compassion	− 1.47	0.35	− 4.16	< .001	.02
Conditional effects					
Self-compassion	*B*	*SE*	*t*	*p*	95% CI
Low	3.34	0.25	13.54	< .001	[2.85, 3.82]
Medium	2.85	0.20	14.07	< .001	[2.45, 3.25]
High	1.69	0.32	5.25	< .001	[1.06, 2.33]

SE = Standard Error. FCV-19S = Fear of COVID-19 Scale

The moderating effect of SC on the relationship between COVID-19 perceived impacts (C19-IS) and depressive symptoms (PHQ-9) was not statistically significant (Table 5). However, self-compassion significantly moderated the relationship between COVID-19 perceived impacts (C19-IS) and anxiety symptoms (GAD-7) (see Table 5 for coefficients and conditional effects). As shown in Fig. 3 (Supplemental Materials), when COVID-19 perceived impacts were high, there was a larger difference in GAD-7 scores between highly self-compassionate individuals and those with moderate or low levels of SC compared to other levels of COVID-19 perceived impacts.

**Table 5:** Moderating effect of self-compassion on relationships of COVID-19 impacts

	*B*	*SE*	*t*	*p*	*Δr*^*2*^
COVID-19 impacts and depression					
Model summary	*F*(3, 346) = 53.75, *p* < .001, *r*^*2*^ = .32				
Constant	7.79	0.31			
C19-IS	0.69	0.14	5.07	< .001	
Self-compassion	− 5.05	0.51	− 9.90	< .001	
C19-IS x self-compassion	− 0.15	0.19	− 0.81	.418	.001
COVID-19 impacts and anxiety					
Model summary	*F*(3, 346) = 67.56, *p* < .001, *r*^*2*^ = .37				
Constant	13.48	0.24			
C19-IS	0.67	0.11	6.39	< .001	
Self-compassion	− 4.19	0.40	− 10.59	< .001	
C19-IS x self-compassion	− 0.31	0.15	− 2.09	.038	.008
Conditional effects					
Self-compassion	*B*	*SE*	*t*	*p*	95% CI
Low	0.80	0.13	6.23	< .001	[0.55, 1.06]
Medium	0.70	0.11	6.50	< .001	[0.49, 0.92]
High	0.46	0.14	3.34	< .001	[0.19, 0.73]

*Note. SE*, standard error; *C19-IS *= COVID-19 Impact Scale

## Discussion

Study 1 results demonstrated expected relationships between dispositional mindfulness (DM), self-compassion (SC), and COVID-19 stress and emotional impact. Contrary to expectations, scores on the short-form measure of DM (FFMQ-15) did not moderate relationships of COVID-19 fears/impacts and psychological disorder symptoms. Partially consistent with hypotheses, results suggested a moderating effect of SC on the relationships of COVID-19 stress and anxiety symptoms. The buffering effects of SC on symptoms appear to be particularly salient in the context of high levels of COVID-19 stress. In the current study, FFMQ-15 possessed McDonald’s Omega that was below recommended cut-offs (*ω* = 0.7; Dunn et al., 2014), suggesting potential issues with internal consistency. It is noteworthy that brief scales by their very nature tend to assess constructs using narrower operational definitions. Second, we assessed the emotional impact of COVID-19 using a bespoke, never before validated measure, and so could not account for measurement issues.

## Study 2

In Study 2, and in the context of Study 1 results, we sought to evaluate the buffering hypothesis of DM as assessed by more than only one validated measure of the construct—the original 39-item FFMQ (Baer et al., 2006), and the Mindful Attention and Awareness Scale (MAAS; Brown & Ryan, 2003). Second, we sought to assess participants’ COVID-19 stress with the recently developed COVID Stress Scales (CSS; Taylor et al., 2020a), which measure COVID-19 stress along five dimensions. Despite its recent development, the CSS has been validated across several samples, and across cultures and languages (Taylor, 2021). We hypothesized that scores on DM measures (MAAS; FFMQ-39) would (H2a) significantly and negatively correlate with COVID-19 stress, and depression and anxiety symptom severity; and (H2b) moderate the relationship between COVID-19 stress and psychological disorder symptoms.

## Method

### Participants

Five hundred and fifty-seven respondents were initially recruited from Amazon’s Mechanical Turk (MTurk). A total of 19 respondents were excluded from analyses for missing data exceeding 19% of scale items (*n* = 11) and failing attention check items (*n* = 8). The final sample included 538 participants. *n* = 295 identified as cis men, *n* = 234 as cis women, *n* = 5 as non-binary/third gender, *n* = 2 as trans men, *n* = 1 as trans woman, and *n* = 1 preferred not to say; *M*_*age*_ = 40.00, *SD*_*age*_ = 11.82). Refer to Table 1 for a summary of pertinent demographic variables. Participants were compensated $3.00 USD for their participation in the study. This study was approved by the University of Regina’s Research Ethics Board (#2021–172). Data collection took place in February 2022.

### Procedures

After providing consent, participants completed the FFMQ-39, MAAS, CSS, PHQ-9, and GAD-7 in a random order. Participants then completed the demographic items. At the end of the survey, participants were thanked, debriefed, and compensated for their participation.

### Measures

Refer to Study 1 for descriptions of PHQ-9 and GAD-7. Measure descriptives, reliabilities, and missing data rates for Study 2 are presented in Table 6.

**Table 6:** Study 2 measures descriptive statistics, reliabilities, and missing data rates

	Mean (SD)	Cronbach alpha (*α*)	McDonald’s omega (ω)	Missing data rate (%)
PHQ-9 (/27)	6.25 (6.37)	.92	.92	0.00
GAD-7 (/21)	5.32 (5.46)	.93	.94	0.00
FFMQ-39 (195)	138.10 (25.08)	.94	.93	0.00
MAAS (/6)	4.37 (1.08)	.95	.95	0.00
CSS-DAN (/48)	14.53 (11.83)	.95	.95	3.78
CSS-SEC (/24)	4.83 (5.93)	.92	.92	0.00
CSS-CHE (/24)	4.83 (5.61)	.91	.91	0.00
CSS-TSS (/24)	3.59 (5.38)	.95	.95	0.00

*PHQ-9*, Patient Health Questionnaire – 9; *GAD-7*, Generalized Anxiety Disorder – 7; *FFMQ-39*, Five Factor Mindfulness Questionnaire – 39; *MAAS*, Mindful Attention Awareness Scale; *CSS-DAN*, COVID-19-related danger and contamination fears; *CSS-SEC*, COVID-19-related fears about economic consequences; *CSS-CHE*, COVID-19-related compulsive checking and reassurance seeking; *CSS-TSS*, COVID-19-related traumatic stress symptoms; *SD*, standard deviation

#### Five-Facet Mindfulness Questionnaire

*The Five-Facet Mindfulness Questionnaire (FFMQ-39;* Baer et al., 2006) is the original, 39-item version of the FFMQ-15 (Baer et al., 2008). The scale follows the same format as FFMQ-15: participants are asked to rate statements on a Likert scale ranging from 1 (*Never or very rarely true)* to 5 (*Very often or always true)*. Furthermore, like FFMQ-15, it also measures five elements of mindfulness: Description, Observation, Acting with Awareness, Nonjudging, and Nonreactivity. Total scores represent dispositional mindfulness. Higher scores reflect greater levels of dispositional mindfulness. Previous studies have established that FFMQ-39 is a valid and reliable measure of mindfulness (Christopher et al., 2012). FFMQ-39 has also been found to be more reliable than its short forms in measuring higher levels of latent mindfulness (Pelham III et al., 2019). In the current study, FFMQ-39 showed excellent internal consistency.

#### Mindful Attention Awareness Scale

*The Mindful Awareness Attention Scale (MAAS;* Brown & Ryan, 2003*)* is a 15-item scale used to measure dispositional mindfulness. Participants are asked to rate statements about their everyday experience (e.g., “I could be experiencing some emotion and not be conscious of it until some time later”) on a 6-point Likert scale ranging from 1 (*Almost Always)* to 6 *(Almost Never).* Dispositional mindfulness is derived by computing the mean of all items. Higher scores represent greater levels of dispositional mindfulness. Support for the psychometric properties of MAAS has been mixed (Osman et al., 2016); however, in the current study, MAAS showed excellent internal consistency.

#### The COVID Stress Scales

*The COVID Stress Scales (CSS;* Taylor et al., 2020a*)* are a 36-item measure of COVID-19-related worries, experiences, and behaviours engaged in during the last seven days. The CSS contains five subscales: Danger and contamination fears, fears about economic consequences, xenophobia, compulsive checking and reassurance seeking, and traumatic stress symptoms. Participants rate statements (e.g., “I am worried about catching the virus”) on a 5-point Likert scale ranging from 0 (*Not at all* or *Never)* to 4 (*Extremely* or *Almost Always).* For the purpose of this study, the xenophobia subscale was omitted from the analyses. Data for the development of the CSS was collected in March 2020, shortly after COVID-19 was declared a pandemic. In this early stage of the pandemic, there was an increase in xenophobic attitudes, particularly towards Asians (Reny & Barreto, 2022). However, given the extent to which the pandemic has evolved since 2020, and the content of the xenophobia items (e.g., “If I went to a restaurant that specialized in foreign foods, I’d be worried about catching the virus”), the xenophobia subscale was not deemed relevant. Previous studies have found support for the psychometric properties of the CSS (Taylor et al., 2020a, b). Scores on the CSS have also been found to be related to psychological disorder symptoms and behaviours such as panic buying and avoiding public transport and grocery stores (Taylor et al., 2020b). The four subscales showed excellent internal consistency in the current study.

### Data Analyses

Data analysis steps for Study 2 were consistent with the analytic steps conducted in Study 1. We examined H2a using Pearson product-moment correlation analysis between scores on the CSS, FFMQ-39, MAAS, PHQ-9, and GAD-7. We used Hayes’ (2017) PROCESS SPSS macro (model 1) to the moderation effects (H2b) of FFMQ-39 and MAAS scores in the relationships of each of the CSS subscales with psychological disorder symptom measures. We used Bonferroni corrections for the moderation analyses. Accordingly, findings were considered statistically significant at alpha = 0.5/4 = 0.0125.

## Results

### Preliminary Data Checks

Skewness ranged from − 0.66 (MAAS) to 1.12 (CSS-SEC), and kurtosis ranged from -0.53 (CSS-DAN) to 0.26 (MAAS). These values were all within acceptable ranges (Cain et al., 2017). Harman’s single-factor test was not significant, accounting for only 29.29% of the variance.

### Zero-Order Correlations

Zero-order correlations between COVID-19-related stress, DM (FFMQ-39; MAAS), and psychological disorder symptoms are presented in Table 7. FFMQ-39 scores and MAAS scores correlated negatively with depressive symptoms (PHQ-9), anxiety symptoms (GAD-7), and scores on each of the COVID-19 stress scales. Mindful describing (FFMQ-Des), attention to awareness (FFMQ-AA) and non-judging (FFMQ-NJ) correlated negatively with COVID-19-related danger and contamination fears (CSS-DAN), fears about economic consequences (CSS-SEC), traumatic stress symptoms (CSS-TSS) and compulsive checking and reassurance seeking (CSS-CHE). Non-reactivity (FFMQ-NR) correlated negatively with COVID-19-related danger and contamination fears, depressive symptoms, and anxiety symptoms. Finally, mindful observation (FFMQ-Obs) did not correlate with COVID-19-related stress or psychological disorder symptoms.

**Table 7:** Study 2 correlations between COVID-19 cognitions, emotional regulation strategies, and mental health symptoms

	CSS-DAN	CSS-SEC	CSS-CHE	CSS-TSS
PHQ-9	.48***	.51***	.39***	.59***
GAD-7	.52***	.51***	.36***	.61***
MAAS	− .18***	− .18***	− .14***	− .18***
FFMQ-39	− .30***	− .29***	− .23***	− .38***
FFMQ-Obs	.08	.07	.08	.03
FFMQ-Des	− .18***	− .18***	− .15***	− .25**
FFMQ-AA	− .35***	− .35***	− .31***	− .46***
FFMQ-NJ	− .39***	− .40***	− .36***	− .50***
FFMQ-NR	− .13**	− .05	− .01	− .05

*PHQ-9*, Patient Health Questionnaire – 9; *GAD-7*, Generalized Anxiety Disorder – 7; *FFMQ-39*, Five Factor Mindfulness Questionnaire – 39; *MAAS*, Mindful Attention Awareness Scale; *CSS-DAN*, COVID-19-related danger and contamination fears; *CSS-SEC*, COVID-19-related fears about economic consequences; *CSS-CHE*, COVID-19-related compulsive checking and reassurance seeking; *CSS-TSS*, COVID-19-related traumatic stress symptoms

^**^p < .01

^***^p $$\le$$ .001

### Moderating Effect of Mindfulness on CSS and Psychological Disorder Symptoms

The moderating effects of mindfulness as measured by FFMQ-39 and MAAS on the relationships between COVID-19-related stress and depression and anxiety symptoms are presented in Table 7 and Tables S2 and S3 (Supplementary Materials). The moderating effect of DM measured by MAAS was not statistically significant for the relationships between COVID-19-related stress and psychological disorder symptoms. The moderating effect of mindfulness measured by FFMQ-39 was also not statistically significant for the relationships between COVID-19-related traumatic stress symptoms and psychological disorder symptoms.

FFMQ-39 significantly moderated the relationships of COVID-19-related danger and contamination fears, fears about economic consequences, and compulsive checking and reassurance seeking, with depression and anxiety symptoms. For each relationship, higher levels of COVID-19-related stress were associated with fewer depressive and anxious symptoms at higher levels of FFMQ-39-measured mindfulness (see Supplemental Material 1 Figs. 4 to 9). Conditional effects are presented in Tables 8 and 9.

**Table 8:** Moderating effect of FFMQ-39 on the relationships of COVID-19 stress and depression

	*B*	*SE*	*t*	*p*	*Δr*^*2*^
CSS-TSS and depression					
Model summary:	*F*(3, 534) = 169.31, *p* < .001, *R*^*2*^ = .70				
Constant	17.58	1.38			
CSS-TSS	.93	.26	3.64	< .001	
FFMQ-39	− .10	.01	− 9.95	< .001	
CSS-TSS x FFMQ-39	− .003	.002	− 1.65	.101	.003
CSS-DAN and depression					
Model summary:	*F*(3, 534) = 150.21, *p* < .001, *R*^*2*^ = .46				
Constant	13.27	1.81			
CSS-DAN	.64	.09	7.17	< .001	
FFMQ-39	− .07	.012	− 5.71	< .001	
CSS-DAN x FFMQ-39	− .003	.001	− 5.23	< .001	.03
Conditional effects					
FFMQ-39	*B*	*SE*	*t*	*p*	95% CI
Low	.25	.02	11.36	< .001	[.21, .30]
Medium	.17	.02	9.63	< .001	[.14, .21]
High	.08	.03	2.86	.004	[.02, .13]
CSS-SEC and depression					
	*B*	*SE*	*t*	*p*	*Δr*^*2*^
Model summary:	*F*(3, 534) = 160.56, *p* < .001, *R*^*2*^ = .47				
Constant	16.10	1.54			
CSS-SEC	1.30	.20	6.60	< .001	
FFMQ-39	− .09	.01	− 8.08	< .001	
CSS-SEC x FFMQ-39	− .01	.002	− 4.61	< .001	.02
Conditional effects					
FFMQ-39	*B*	*SE*	*t*	*p*	95% CI
Low	.53	.04	11.96	< .001	[.43, .61]
Medium	.37	.04	10.30	< .001	[.30, .44]
High	.18	.06	3.01	.003	[.06, .30]
CSS-CHE and depression					
	*B*	*SE*	*t*	*p*	*Δr*^*2*^
Model summary:	*F*(3, 534) = 125.09, *p* < .001, *R*^*2*^ = .41				
Constant	18.32	1.61			
CSS-CHE	1.23	.22	5.53	< .001	
FFMQ-39	− .10	.01	− 8.78	< .001	
CSS-CHE x FFMQ-39	− .01	.002	− 4.20	< .001	.02
Conditional effects					
FFMQ-39	*B*	*SE*	*t*	*p*	95% CI
Low	.43	.05	8.89	< .001	[.34, .53]
Medium	.27	.04	6.90	< .001	[.20, .35]
High	.08	.07	1.16	.247	[-.06, .21]

*FFMQ-39*, Five factor mindfulness questionnaire—39; *CSS-DAN*, COVID-19-related danger and contamination fears; *CSS-SEC*, COVID-19-related fears about economic consequences; CSS-CHE, COVID-19-related compulsive checking and reassurance seeking; *CSS-TSS*, COVID-19-related traumatic stress symptoms; *SE* standard error

**Table 9:** Moderating effect of FFMQ-39 on the relationships of COVID-19 stress and anxiety

	*B*	*SE*	*t*	*p*	*Δr*^*2*^
CSS-TSS and anxiety					
Model summary	*F*(3, 534) = 179.90, *p* < .001, *R*^*2*^ = .71				
Constant	15.85	1.17			
CSS-TSS	.38	.22	1.75	.080	
FFMQ-39	− .09	.01	− 10.92	< .001	
CSS-TSS x FFMQ-39	.001	.002	.38	.703	.0001
CSS-DAN and anxiety					
Model summary	*F*(3, 534) = 163.50, *p* < .001, *R*^*2*^ = .48				
Constant	12.10	1.52			
CSS-DAN	.48	.08	6.37	< .001	
FFMQ-39	− .07	.01	− 6.52	< .001	
CSS-DAN x FFMQ-39	− .002	.001	− 4.10	< .001	.02
Conditional effects					
FFMQ-39	*B*	SE	*t*	*p*	95% CI
Low	.22	.02	11.94	< .001	[.19, .26]
Medium	.17	.02	11.29	< .001	[.14, .20]
High	.11	.02	4.74	< .001	[.06, .15]
CSS-SEC and anxiety					
	*B*	*SE*	*t*	*p*	*Δr*^*2*^
Model summary	*F*(3, 534) = 159.65, *p* < .001, *R*^*2*^ = .47				
Constant	14.25	1.32			
CSS-SEC	1.04	.17	6.11	< .001	
FFMQ-39	− .08	.01	− 8.44	< .001	
CSS-SEC x FFMQ-39	− .01	.001	− 4.14	< .001	.02
Conditional effects					
FFMQ-39	*B*	SE	*t*	*p*	95% CI
Low	.44	.04	11.64	< .001	[.37, .51]
Medium	.32	.03	10.35	< .001	[.26, .38]
High	.17	.05	3.37	.001	[.07, .27]
CSS-CHE and anxiety					
	*B*	*SE*	*t*	*p*	*Δr*^*2*^
Model summary	*F*(3, 534) = 115.56, *p* < .001, *R*^*2*^ = .39				
Constant	16.96	1.40			
CSS-CHE	.83	.19	4.29	< .001	
FFMQ-39	− .09	.01	− 9.50	< .001	
CSS-CHE x FFMQ-39	− .005	.001	− 3.12	.002	.01
Conditional effects					
FFMQ-39	*B*	SE	*t*	*p*	95% CI
Low	.32	.04	7.47	< .001	[.23, .40]
Medium	.21	.03	6.20	< .001	[.15, .28]
High	.09	.06	1.50	.135	[− .03, .20]

*FFMQ-39*, Five factor mindfulness questionnaire-—39; *CSS-DAN*, COVID-19-related danger and contamination fears; *CSS-SEC*, COVID-19-related fears about economic consequences; *CSS-CHE*, COVID-19-related compulsive checking and reassurance seeking; *CSS-TSS*, COVID-19-related traumatic stress symptoms; *SE*, standard error

## Discussion

Study 2 results revealed the expected relationships between DM and its facets as measured by the MAAS and the original version of the FFMQ, and COVID-19 stresses. Contrary to hypotheses, scores on MAAS did not appear to moderate the relationships of COVID-19 stresses and symptoms of psychological disorders. However, and consistent with hypotheses, scores on FFMQ-39 moderated the relationships between COVID-19 stresses (e.g., danger and contamination fears; compulsive checking/reassurance seeking; fears about economic consequences) and symptoms of psychological disorders. This suggested FFMQ and MAAS may be assessing heterogenous aspects of DM that have differential mental health effects.

## General Discussion

In the current investigation, we examined direct and moderating relationships between DM and SC, COVID-19-related stress and impacts, and psychological disorder symptoms. To the authors’ knowledge, this is one of few studies of the buffering effects of DM and SC in the relationship between COVID-19 stress and impacts and symptoms of psychopathology. Mental health research has focused disproportionality on risk mechanisms (Masten, 2001), while neglecting the potential role of protective mechanisms and resilience in disrupting cycles of pathology. This is important since crises such as COVID-19 evoke a slew of unconditioned, naturalistic fears in most people, that are evolutionarily designed to confer resources to manage threats. The cultivation of a protective mechanism, such as those associated with DM and SC, that act to attenuate the deleterious effects of risk factors may be a cost-effective and practical approach. Our results provide preliminary evidence of the viability of this approach during COVID-19.

Consistent with our hypotheses, we found that both depression and anxiety symptoms were positively and moderately correlated with increased COVID-related fears, stress, and perceptions of impact. This result is consistent with several lines of research that demonstrate that COVID-19 fears are associated with increased symptoms of psychopathology (Wu et al., 2021). Furthermore, we found DM and SC negatively correlated with COVID-19 fears, stress, and perceptions of impact. This is unsurprising and fits with the portrait painted by previous literature on the correlates of these protective factors. That is, researchers have shown that both DM and SC are consistently and negatively associated with psychological disorder symptoms Conversano et al., 2020; Dillard & Meier, 2021; Kavakli et al., 2020; Matos et al., 2022).

In Study 1, and contrary to our hypotheses, we did not find that DM as assessed by the 15-item FFMQ significantly moderated the relationships between COVID-19 fears, impacts, and symptoms of depression or anxiety; however, in Study 2, we found scores on the original 39-item FFMQ significantly moderated the relationships of COVID-19 stress and psychological disorder symptoms. Surprisingly, this significant buffering effect disappeared when scores on the MAAS were entered as the moderator. There are several potential explanations for this pattern of results. First, the FFMQ-15 evidenced relatively low internal consistencies in Study 1, as demonstrated by a low Cronbach’s alpha, and more critically, a low McDonald’s omega (Kalkbrenner, 2021; McDonald, 1999). This suggested that the higher-order, general mindfulness factor may not be a significant driver of scores on items corresponding to each of the five lower-order facets composing the scale (Davenport et al., 2015). The mechanisms driving the effects of DM likely rely less on the simple summation of capacities across the five facets, and more on their synergistic or interactional effects. It is through this synergistic bond between the components of mindfulness, which can otherwise be understood as the same as a general mindfulness factor, that DM is conceptualized to exact its buffering effects (Lindsay & Creswell, 2017; Shapiro et al., 2006).

This gestalt (or the “whole is greater than the sum of its parts”) conceptualization of the mechanisms of DM is supported by several lines of evidence. First, researchers have consistently found positive associations between DM, attentional, emotional, and general self-regulation, nonreactivity, and emotional stability (Tomlinson et al., 2018). Second, brain imaging studies suggest DM is simultaneously correlated with (a) higher neural activation of brain regions associated with self-regulation, and (b) lower neural activation of regions corresponding with emotional reactivity (Rau & Williams, 2016). Finally, interactions between differing facets of DM (e.g., interaction between Observing and Nonjudgment as measured by the FFMQ) appear to differentially predict symptoms of psychological disorders (Eisenlohr-Moul et al., 2012).

It is plausible that a measure that fails to capture a general mindfulness factor driving scores on individual facets—as demonstrated by the FFMQ-15 in Study 1—is also unlikely to demonstrate moderation. In a recent study employing latent profile analysis, researchers found only 68% of respondents completing the short form of the FFMQ demonstrated a “general mindfulness” profile (Lecuona et al., 2022). Two other respondent profiles emerged, including a "Non-judgementally Aware” profile (24.8%), typified by a non-discerning form of awareness which does not fit a typical high mindfulness profile, and a “Judgmentally Observing” profile (7.4%). This pattern of findings suggests that the interactions between awareness, non-reactivity, and non-judgement is key to a true “general mindfulness” profile and hence to understanding the mechanisms of DM (Rau & Williams, 2016).

Study 2 findings did not support our hypothesis regarding the moderation effects of DM assessed by the MAAS (Brown & Ryan, 2003) in the relationship between COVID-19 stress and symptoms of psychological disorders. This is inconsistent with previous findings (Conversano et al., 2020) and suggests the FFMQ and MAAS are assessing differing aspects of mindfulness, which is consistent with how they were conceptualized and developed (Hanley & Garland, 2017). While the MAAS defines mindfulness more narrowly as present-moment awareness (Brown & Ryan, 2003), FFMQ’s definition is more broad and representative, drawing in nonjudgement and nonreactivity as critical components (Baer et al., 2006). As discussed above, the synergy between the parts of DM is likely what is driving its effects, and hence is more likely captured by an internally consistent, multidimensional measure of the construct (e.g., FFMQ).

Very few studies to date have examined the moderating effects of DM in the relationship of COVID-19 stress and psychological disorder symptoms. Several have demonstrated DM to be negatively correlated with stress and symptoms during the pandemic (e.g., Conversano et al., 2020). There are several notable differences between extant literature on mindfulness during the COVID-19 crisis and the present investigation. First, while researchers leading studies in this area have employed measures of DM such as the Mindfulness Attention Awareness Scale (MAAS; Conversano et al., 2020), and Cognitive and Affective Mindfulness scale (Dillard & Meier, 2021), no studies have used FFMQ to this end. Second, the extant literature more consistently shows the buffering effect of regular mindfulness meditation practice, not necessarily dispositional mindfulness, in buffering the deleterious effects of COVID-19 stress (Zheng et al., 2020). Some researchers have fittingly raised concerns about whether dispositional mindfulness scales are able to accurately assess cultivated forms of mindfulness (Chiesa, 2013; Van Dam et al., 2018). However, evidence consistently demonstrates DM increases as a function of participation in mindfulness-based interventions (Quaglia et al., 2016).

In Study 1, we found that dispositional SC was a significant moderator in the relationships of COVID-related fears and emotional impact, and symptoms of psychopathology. This is consistent with previous literature showing that SC moderates the relationship between negative affective states and psychopathology (Trompetter et al., 2017). The present findings are also in line with new evidence suggesting broad capacities for compassion, whether for self, from self, or from others, buffer the effects of COVID-19 threats on psychological distress across cultures (Matos et al., 2022). The findings of the present study provide support for theories of SC as tapping into care seeking, caregiving, and nurturing capacities (Hermanto & Zuroff, 2016). That is, and consistent with evolutionary conceptualizations of compassion, SC appears to offer protection from suffering in the form of attenuated relationships of stress and symptoms of psychopathology (Goetz et al., 2010). Consistent with these hypotheses, we demonstrated that high levels of SC mitigated the effects of elevated levels of COVID-19 fears and perceived impacts.

The current study possessed several strengths and thus contributes in a meaningful way to this growing literature. First, while there are several published studies demonstrating the relationships between DM *or* SC and COVID-19 stress and fears, no studies to date have compared the relationships of both constructs on pandemic-related psychosocial outcomes. Second, there are no published studies to date directly comparing the differential buffering effects of DM as assessed by commonly used scales of the construct. Furthermore, the authors are aware of very few studies to date that have examined the moderating effects of both DM and/or SC in the relationships of COVID-19 fears, stress, and emotional impact and symptoms of psychopathology.

### Limitations and Future Research

The study also suffered from several limitations that pave the way for future research in this area. First, while the demographics of crowdsourcing, online samples approximate those of adults in the general population, studies have found notable differences compared to general population samples collected through other means (with online samples being younger, more educated, and having higher self-reported symptoms of psychopathology; Hulland & Miller, 2018). Second, we focused exclusively on dispositional forms of DM and SC, and as mentioned earlier, these can depart markedly from cultivated forms of these capacities (Rau & Williams, 2016), Third, and relatedly, items of the FFMQ have been demonstrated to differentially operate among meditators and non-meditators, casting doubt over its cross-group equivalence (Van Dam et al., 2009). Fourth, the cross-sectional methodology limits any inferences to causality. Finally, and given that we assessed all target constructs online using self-report measures, common method variance may have worked to inflate relationships among the measured variables, and hence produced spurious or unreliable results (Podsakoff et al., 2012).

These limitations open the door for several additional future investigations. Future research should examine whether the buffering effect of SC can be replicated with other measures of the construct, and with more diverse populations. Second, future research should examine whether cultivated forms of mindfulness may offer a better buffer against the effects of COVID-19 stress in depression and anxiety. This is especially true given the finding that mindfulness scores were more strongly associated with COVID-19 fears than were those of SC. This could point to the potentially mediating (in addition to moderation) effect of DM in the relationship of COVID-19 fears, stress, and symptoms of psychopathology.

Our study replicated the buffering effects of DM and SC in mitigating the effects of COVID-19 fears, stress, and emotional impacts on symptoms of depression and anxiety. This result needs to be replicated with more diverse samples, and cultivated forms of DM and SC need to be tested appropriately using randomized controlled trials. However, given the malleability of these protective dispositional factors even in the context of briefer, self-guided interventions (Beshai et al., 2020), the results of the current study offer hope to millions still struggling with COVID-19 fears and stress, and their devastating effects on mental health.

## Supplementary Information

Below is the link to the electronic supplementary material.

Supplementary file1 (DOCX 10083 KB)

## Data Availability

All data are available at the Open Science Framework (http://doi.org/10.17605/OSF.IO/E6ZWH).
