# Supplementary material for: Dispositional Mindfulness and Self-Compassion Buffer the Effects of COVID-19 Stress on Depression and Anxiety Symptoms
Source: Mindfulness (N Y). 2022 Oct 22;13(12):3028–42. doi: 10.1007/s12671-022-02008-0 (PMC9589785; doi:10.1007/s12671-022-02008-0)
Supplement: Supplementary file 1 — Supplementary file1 (DOCX 10083 KB) [file 12671_2022_2008_MOESM1_ESM.docx]

**Dispositional Mindfulness and Self-Compassion buffer the Effects of COVID-19 Stress on Depression and Anxiety Symptoms**

***Mindfulness***

**Table S1.** *Moderating effect of FFMQ-15*

|  | *B* | *SE* | *t* | *p* | *Δr^2^* |
| --- | --- | --- | --- | --- | --- |
| COVID-19 fears and depression | | | | | |
| **Model Summary:** | ***F*(3, 346) = 102.32, *p* < .001, *r^2^* = .47** | | | | |
| Constant | 7.68 | 0.29 |  |  |  |
| FCV-19S | 3.11 | 0.29 | 10.90 | <.001 |  |
| Mindfulness | -0.37 | 0.05 | -7.85 | <.001 |  |
| FCV-19S x Mindfulness | -0.06 | 0.04 | -1.33 | .185 | .003 |
| COVID-19 fears and anxiety | | | | | |
| **Model Summary:** | ***F*(3, 346) = 97.81, *p* < .001, *r^2^* = .68** | | | | |
| Constant | 13.57 | 0.24 |  |  |  |
| FCV-19S | 2.76 | 0.23 | 11.88 | <.001 |  |
| Mindfulness | -0.23 | 0.04 | -6.06 | <.001 |  |
| FCV-19S x Mindfulness | -0.01 | 0.04 | -0.30 | .767 | .0001 |
| COVID-19 emotional impacts and depression | | | | | |
| **Model Summary:** | ***F*(3, 346) = 61.93, *p* < .001, *r^2^* = .35** | | | | |
| Constant | 7.81 | 0.30 |  |  |  |
| COVID-19 Impact | 0.77 | 0.13 | 5.83 | <.001 |  |
| Mindfulness | -0.51 | 0.05 | -10.93 | <.001 |  |
| COVID-19 Impact x Mindfulness | -0.01 | 0.02 | -0.57 | .569 | .0006 |
| COVID-19 emotional impacts and anxiety | | | | | |
| **Model Summary:** | ***F*(3, 346) = 57.80, *p* < .001, *r^2^* = .33** | | | | |
| Constant | 13.58 | 0.24 |  |  |  |
| COVID-19 Impact | 0.75 | 0.11 | 7.00 | <.001 |  |
| Mindfulness | -0.37 | 0.04 | -9.53 | <.001 |  |
| COVID-19 Impact x Mindfulness | -0.007 | 0.02 | -0.43 | .669 | .0004 |

*Note.* SE = Standard Error. FFMQ-15 = Five Factor Mindfulness Questionnaire-15, FCV-19S = Fear of COVID-19 Scale.

**Table S2.** *Moderating effect of MAAS on the relationship between COVID-19-related stress and depression (PHQ-9).*

|  | *B* | *SE* | *t* | *p* | *Δr^2^* |
| --- | --- | --- | --- | --- | --- |
| **Model Summary:** | ***F*(3, 534) = 77.76, *p* < .001, *R^2^* = .30** | | | | |
| Constant | 7.68 | 1.47 |  |  |  |
| CSS-DAN | .38 | 0.8 | 4.91 | <.001 |  |
| MAAS | -1.12 | 0.31 | -3.57 | <.001 |  |
| CSS-DAN x MAAS | -0.3 | 0.02 | -1.95 | .051 | .01 |
| **Model Summary:** | ***F*(3, 534) = 85.17, *p* < .001, *R^2^* = .32** | | | | |
| Constant | 10.80 | 1.25 |  |  |  |
| CSS-SEC | .44 | .16 | 2.82 | .005 |  |
| MAAS | -1.58 | .27 | -5.89 | <.001 |  |
| CSS-SEC x MAAS | .01 | .04 | .38 | .705 | .0002 |
| **Model Summary:** | ***F*(3, 534) = 55.42, *p* < .001, *R^2^* = .24** | | | | |
| Constant | 12.16 | 1.37 |  |  |  |
| CSS-CHE | .34 | .18 | 1.90 | .059 |  |
| MAAS | -1.79 | .29 | -6.07 | <.001 |  |
| CSS-CHE x MAAS | .01 | .04 | .31 | .758 | .0001 |
| **Model Summary:** | ***F*(3, 534) = 120.21 , *p* < .001, *R^2^* = .40** | | | | |
| Constant | 10.46 | 1.10 |  |  |  |
| CSS-TSS | .55 | .18 | 3.11 | .002 |  |
| MAAS | -1.49 | .23 | -6.55 | <.001 |  |
| CSS-TSS x MAAS | .02 | .04 | .56 | .575 | .0004 |

*Note.* PHQ-9 = Patient Health Questionnaire – 9; MAAS = Mindful Attention Awareness Scale; CSS-DAN = COVID-19-related danger and contamination fears; CSS-SEC = COVID-19-related fears about economic consequences; CSS-CHE = COVID-19-related compulsive checking and reassurance seeking; CSS-TSS = COVID-19-related traumatic stress symptoms.

SE = Standard Error.

**Table S3.** *Moderating effect of MAAS on the relationship between COVID-19-related stress and anxiety (GAD-7).*

|  | *B* | *SE* | *t* | *p* | *Δr^2^* |
| --- | --- | --- | --- | --- | --- |
| **Model Summary:** | ***F*(3, 534) = 86.76, *p* < .001, *R^2^* = .33** | | | | |
| Constant | 6.96 | 1.24 |  |  |  |
| CSS-DAN | .25 | .07 | 3.86 | <.001 |  |
| MAAS | -1.11 | .26 | -4.23 | <.001 |  |
| CSS-DAN x MAAS | -.007 | .01 | -.48 | .634 | .0003 |
| **Model Summary:** | ***F*(3, 534) = 82.16, *p* < .001, *R^2^* = .32** | | | | |
| Constant | 9.20 | 1.08 |  |  |  |
| CSS-SEC | .30 | .13 | 2.20 | .028 |  |
| MAAS | -1.36 | .23 | -5.85 | <.001 |  |
| CSS-SEC x MAAS | .03 | .03 | 1.03 | .306 | .001 |
| **Model Summary:** | ***F*(3, 534) = 47.42, *p* < .001, *R^2^* = .21** | | | | |
| Constant | 10.62 | 1.19 |  |  |  |
| CSS-CHE | .18 | .16 | 1.13 | .259 |  |
| MAAS | -1.56 | .26 | -6.06 | <.001 |  |
| CSS-CHE x MAAS | .03 | .04 | .90 | .370 | .001 |
| **Model Summary:** | ***F*(3, 534) = 127.54 , *p* < .001, *R^2^* = .42** | | | | |
| Constant | 8.91 | .89 |  |  |  |
| CSS-TSS | .30 | .15 | 2.00 | .047 |  |
| MAAS | -1.29 | .19 | -6.69 | <.001 |  |
| CSS-TSS x MAAS | .07 | .03 | 1.90 | .059 | .0039 |

*Note.* GAD-7 = Generalized Anxiety Disorder-7; MAAS = Mindful Attention Awareness Scale; CSS-DAN = COVID-19-related danger and contamination fears; CSS-SEC = COVID-19-related fears about economic consequences; CSS-CHE = COVID-19-related compulsive checking and reassurance seeking; CSS-TSS = COVID-19-related traumatic stress symptoms.

SE = Standard Error.

Visual representation of moderating effects of dispositional mindfulness and self-compassion on the relationships between COVID-19-related variables and depression/anxiety.

**Figure 1.** *Moderating effect of self-compassion on the relationship between COVID-19 fears and depression.*


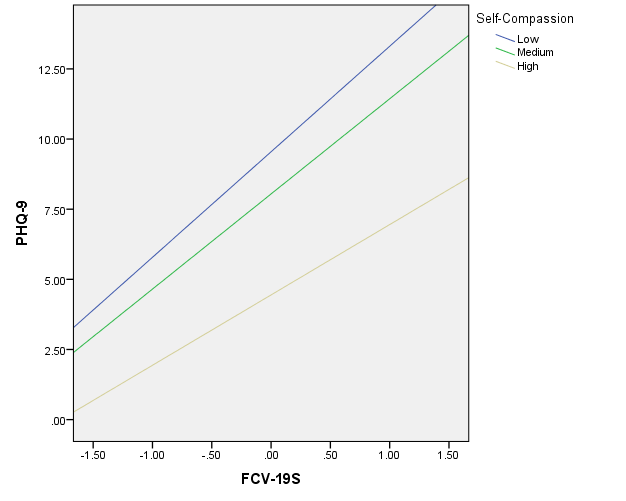


*Note.* PHQ-9 = Patient Health Questionnaire – 9, FCV-19S = Fear of COVID-19 Scale.

**Figure 2.** *Moderating effect of self-compassion on the relationship between COVID-19 fears and anxiety.*


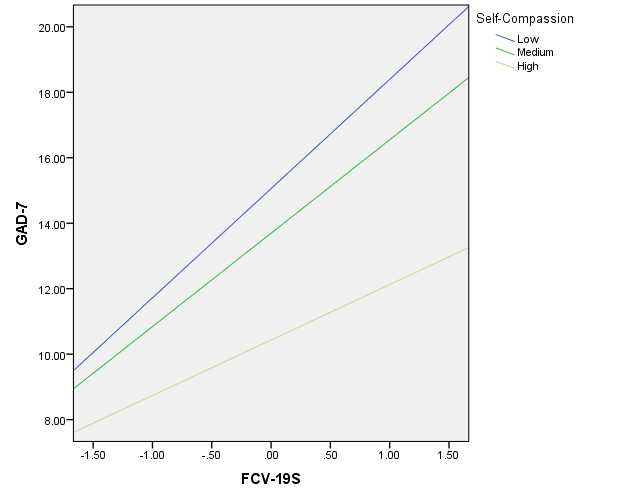


*Note.* GAD-7 = Generalized Anxiety Disorder – 7, FCV-19S = Fear of COVID-19 Scale.

**Figure 3.** *Moderating effect of self-compassion on the relationship between COVID-19 emotional impact and anxiety.*


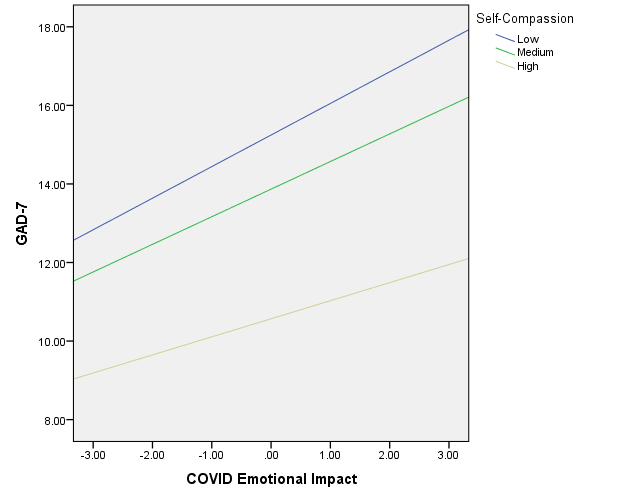


*Note.* GAD-7 = Generalized Anxiety Disorder – 7.

**Figure 4.** *Moderating effect of FFMQ-39 on the relationship between CSS-DAN and depression.*

**
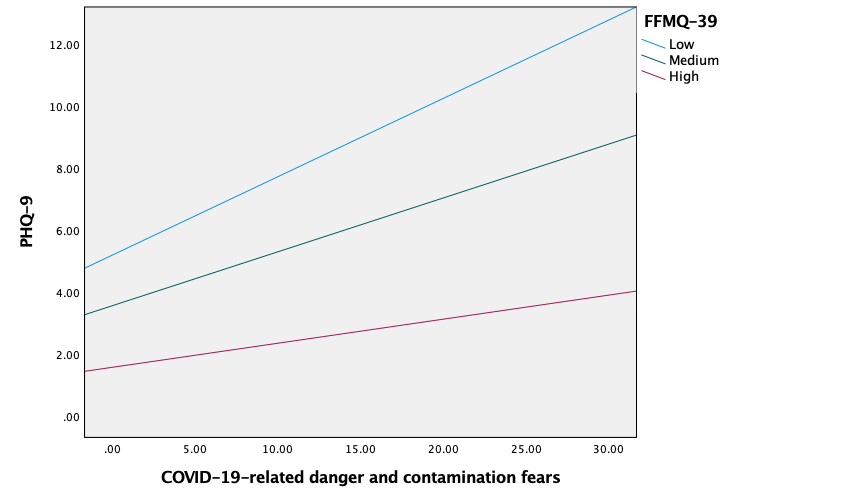
**

*Note.* PHQ – 9 = Patient Health Questionnaire - 9, FFMQ-39 = Five Facet Mindfulness Questionnaire – 39, CSS-DAN = COVID-19-related danger and contamination fears

**Figure 5.** *Moderating effect of FFMQ-39 on the relationship between CSS-SEC and depression.*

*
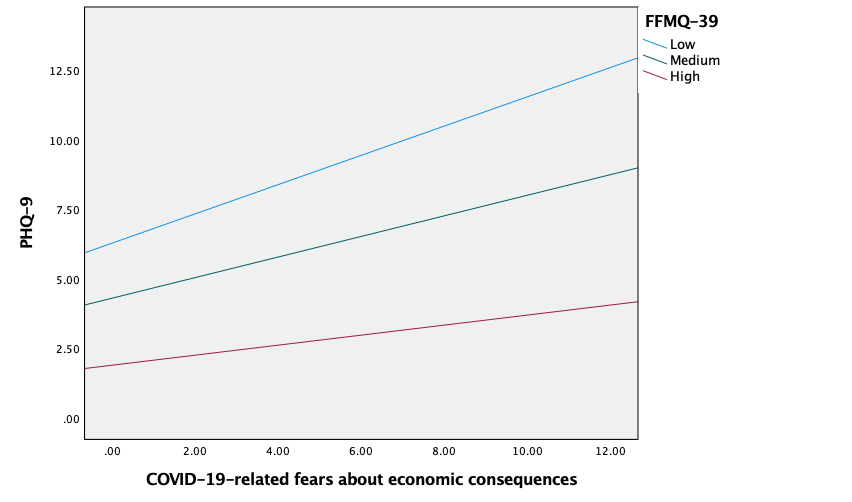
*

*Note.* PHQ – 9 = Patient Health Questionnaire - 9, FFMQ-39 = Five Facet Mindfulness Questionnaire – 39, CSS-SEC = COVID-19-related fears about economic consequences

**Figure 6.** *Moderating effect of FFMQ-39 on the relationship between CSS-CHE and depression.*

**
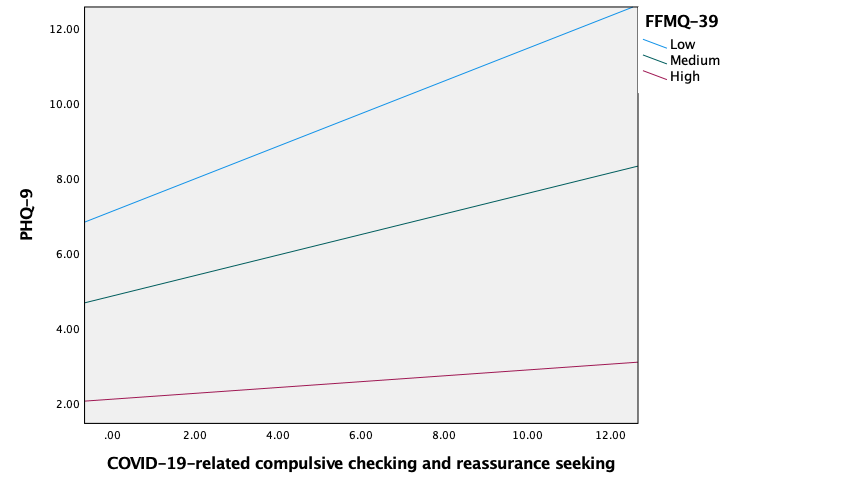
**

*Note.* PHQ – 9 = Patient Health Questionnaire - 9, FFMQ-39 = Five Facet Mindfulness Questionnaire – 39, CSS-CHE = COVID-19-related compulsive checking and reassurance seeking

**Figure 7.** *Moderating effect of FFMQ-39 on the relationship between CSS-DAN and anxiety.*

*
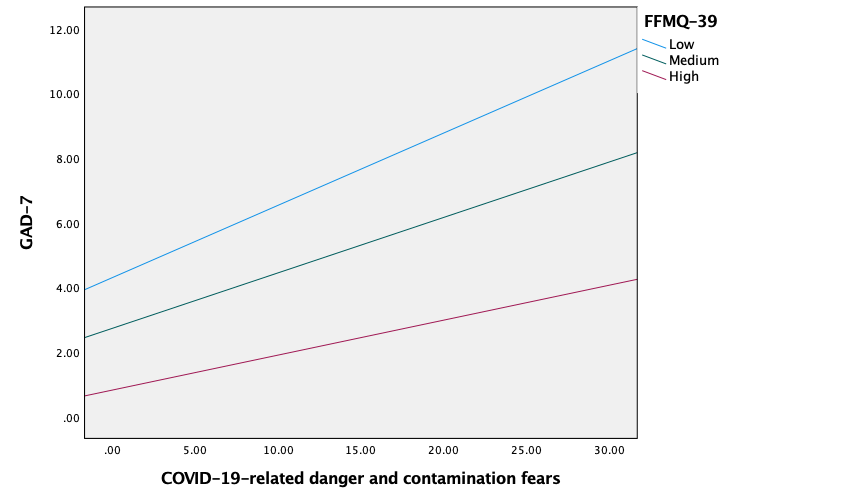
*

*Note.* GAD-7 = Generalized Anxiety Disorder – 7, FFMQ-39 = Five Facet Mindfulness Questionnaire – 39, CSS-DAN = COVID-19-related danger and contamination fears

**Figure 8.** *Moderating effect of FFMQ-39 on the relationship between CSS-SEC and anxiety.*

*
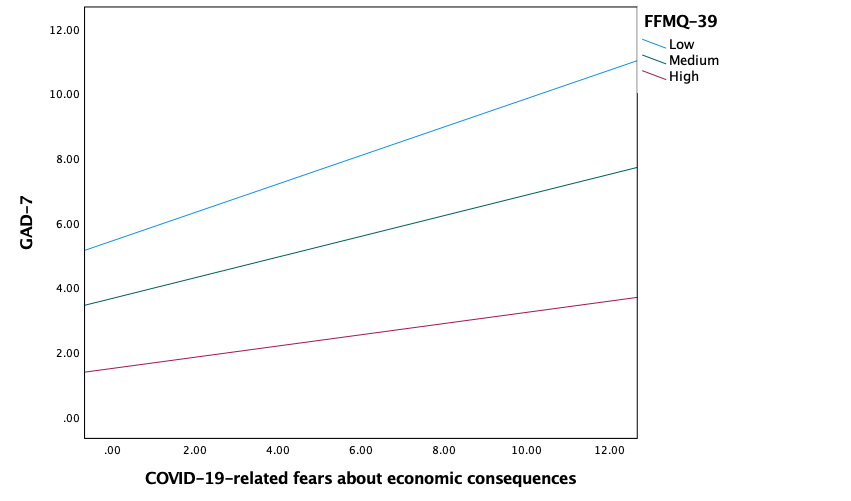
*

*Note.* GAD-7 = Generalized Anxiety Disorder – 7, FFMQ-39 = Five Facet Mindfulness Questionnaire – 39, CSS-SEC = COVID-19-related fears about economic consequences

**Figure 9.** *Moderating effect of FFMQ-39 on the relationship between CSS-CHE and anxiety.*

**
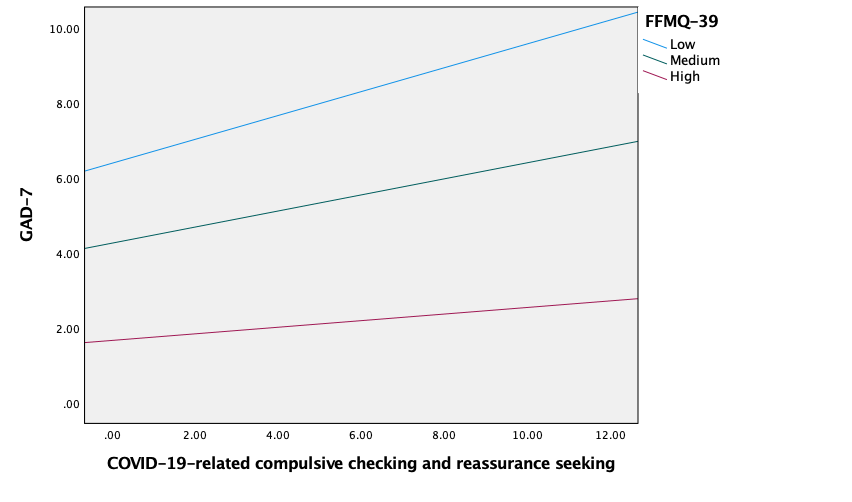
**

*Note.* GAD-7 = Generalized Anxiety Disorder – 7, FFMQ-39 = Five Facet Mindfulness Questionnaire – 39, CSS-CHE = COVID-19-related compulsive checking and reassurance seeking
